# Toward a Commonly Shared Public Policy Perspective for Analyzing Risk Coping Strategies

**DOI:** 10.1111/risa.13505

**Published:** 2020-05-18

**Authors:** Yanwei Li, Araz Taeihagh, Martin de Jong, Andreas Klinke

**Affiliations:** ^1^ Department of Public Administration Nanjing Normal University Nanjing City Jiangsu China; ^2^ Lee Kuan Yew School of Public Policy National University of Singapore Singapore; ^3^ Rotterdam School of Management and Erasmus School of Law Erasmus University Rotterdam Rotterdam The Netherlands; ^4^ Institute for Global Public Policy Fudan University Shanghai China; ^5^ Environmental Policy Institute Memorial University of Newfoundland Corner Brook NL Canada

**Keywords:** Complexity, framework, public policy, review, risk, strategies

## Abstract

The concept of risk has received scholarly attention from a variety of angles in the social, technical, and natural sciences. However, public policy scholars have not yet generated a comprehensive overview, shared understanding and conceptual framework of the main problem‐solving approaches applied by governments in coping with risks. In this regard, our main aim is to examine existing perspectives on prevailing risk coping strategies, find a common denominator among them and contribute to current policy and risk science literature through providing a conceptual framework that systematically spans the spectrum of risk coping strategies and incorporates the essence of the most relevant insights. To this end, we first examine the concept of risk in‐depth by exploring various definitions and types of risk. We then review different approaches proposed by different strands of research for addressing risk. Finally, we assess current knowledge and develop an amalgamated perspective for examining how risks can be addressed by classifying them into six general types of response (no response; prevention; control; precaution; toleration; and adaptation) as well as indicators to identify these responses. We argue that these strategies can function as a heuristic tool for decisionmakers in designing appropriate policies to cope with risks in decision‐making processes.

## 1 INTRODUCTION

Since the early 1970s, risk has been a global buzzword in legislative inquiries, guidance documents, court decisions, workshops, symposia, newspaper and television reports, and published articles (Jasanoff, 1999). The study of risk and uncertainty and the minimization of its impact has received substantial attention from many different academic fields, such as sociology of risk (e.g., Beck, 2009; Lidskog & Sunquist, 2012), governance research (Fisher, 2010; Klinke & Renn, 2012, 2014; Renn, 2008; van Asselt & Renn, 2011; in this special issue), organizational studies (e.g., Perrow, 1999; Pettersen, 2019), communication science (e.g., Friedman, Dunwoody, & Rogers, 1999; Lundgren & McMakin 2018; Morgan, Fischhoff, Bostrom, & Atman, 2002), perception studies (e.g., Burns, Peters, & Slovic, 2012; Siegrist, Gutscher, & Earle, 2005; Slovic, 2000), planning (Fischer, 2009; Fischer & Forester, 1993), project management (Atkinson, Crawford, & Ward, 2006; Chapman & Ward, 2003; Doloi, 2009; Kutsch & Hall, 2010; Osipova & Eriksson, 2013; Rahman & Kumaraswamy, 2004), and complexity sciences (Abid et al., 2014; Biggs et al., 2015; Gerrits, 2016; Taleb, 2012; Walker, Lempert, & Kwakkel,^2013^).

Although a growing number of policy scholars have regarded risk as a key issue in policy processes (Aldrich, 2012; Comfort, Boin, & Demchack, 2010; Nair & Howlett, 2016; Stark, 2014; Taeihagh, 2017; Taeihagh, Givoni, & Bañares‐Alcántara, 2014, 2013), and they all agree that risks pose considerable challenges to decisionmakers in our dynamic and uncertain world (Boin & Lodge, 2016; Duit, 2016; Li, 2016), a comprehensive general review of strategies applied by decisionmakers for coping with risks is hardly available in public policy theories. Risk coping strategies can be viewed as public policy instruments or problem‐solving approaches responding to the challenges and effects of risk. Social science risk research and the policy sciences are lacking a thorough examination: which action plans, policies, and strategies are taken to achieve effective risk management and what the reasons are. In this contribution, we will review and reconstruct the evolution and spectrum of risk coping strategies that are employed as public policy instruments by governments and regulators to master risk issues and minimize their impacts. Risk coping strategies can be viewed as policy instruments responding to the challenges of risk by proving a kind of problem‐solving capacity. To this end, we take a stab to provide a formal examination of the development and whole range of policy‐relevant risk coping strategies from a public policy perspective during the last two decades with the intention of instituting a systematic scheme denominating general categories of risk coping strategies guiding policy analysis to identify and appraise appropriate problem solving. Our aim is to find common ground rather than dissensus among theories and scholars and acquire a conceptual framework that can help in coming to a shared understanding among public policy scholars, interdisciplinary risk scientists, policy makers, and risk practitioners. Furthermore, it can be applied in future empirical research, for instance in comparative cross‐national studies. In this contribution, based on current knowledge, we review prevailing risk coping strategies in public policymaking and synthesize an expanded and advanced understanding of them to assist scholars and practitioners in fully comprehending the spectrum of risk coping strategies.

In the following two sections, we briefly review the current knowledge about the nature of risk and the risk coping strategies primarily derived from relevant academic fields, such as risk governance, public policy, project management and planning, and complexity science. In Section 4, we elaborate on the reframing of risk coping strategies, based on the literature reviewed earlier. Finally, we provide conclusions and implications regarding the application of these risk coping strategies and the future research agendas in Section 5.

## 2 DEFINITION AND TYPES OF RISK

The concept of risk is elusive, contested, and inherently controversial (Borraz, 2011; Fischoff, Watson, & Hope, 1984).^1^ The rise and spectrum of risk coping strategies are closely linked to typical examples or patterns of risk. Its varying definitions and understandings influence the conceptualizations of problem solving in terms of risk coping strategies, especially from the perspective of public policymaking. The definition of risk was first attempted in Knight's classic book, *Risk, uncertainty, and profit*, published in 1921 (Prpić, 2016). In Knight's conception, risk and uncertainty are two highly related concepts. Risk is defined as a measurable uncertainty, while uncertainty is unmeasurable. To make this distinction clearer, Knight proposed that risk can be objectively measured through scientific approaches, whereas uncertainty can be assessed by subjective estimates. Beck (1998) nevertheless uses the terms “risk” and “uncertainty” interchangeably: both refer to scientific and technological consequences.

Walker et al. (2013) do not discuss the differences between uncertainty and risk, but identify five levels of uncertainty that have a stake in the development of risk coping strategies.^2^ Similarly, Stirling (2007) identifies four types of incertitude: *risk, ambiguity, uncertainty*, and *ignorance* (Table I). Risk refers to a situation in which it is not difficult to identify the outcomes and the probabilities of the occurrences of (disastrous) events (Wynne, 1992). Ambiguity^3^ means there is an agreement on the outcomes of a specific event, but there is no knowledge of its probabilities. Uncertainty implies that the probabilities of a specific event are unclear, but there is an agreement on its outcomes.^4^ Ignorance refers to a situation in which both the consequences and likelihood of the occurrences of a specific event are unknown (Collingridge, 1980).

**Table I:** Types of Incertitude (Based on Stirling, 2007)

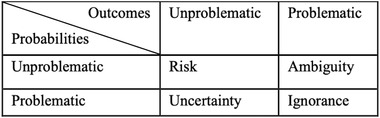

In the late 1990s, Klinke and Renn (2001, 2002) developed a new risk typology for the German Scientific Advisory Council on Global Change (WBGU, 2000), which covers six types of risks, namely, *Sword of Damocles*, *Cyclops*, *Pythia*, *Pandora's box*, *Cassandra*, and *Medusa* (see Table II). The new typology has received considerable attention over time and been applied as template by various government agencies, risk sciences and higher education to order and estimate the similarity or dissimilarity between risk phenomena and reason risk coping strategies and instruments.

**Table II:** Risk Typology (Based on Klinke & Renn, 2001, 2002)

Types of Risk	Description
*Sword of Damocles*	Risks of this type are characterized by low probability, but very high damage potential, such as catastrophes at nuclear plants or chemical facilities.
*Cyclops*	This risk type concerns risks with largely uncertain probabilities of occurrence, but high and relatively well‐known disaster potential, such as earthquakes, volcanic eruptions, or infectious diseases.
*Pythia*	Risks, such as genetic engineering or biological systems engineering are seen as ambiguous, which means probability of occurrence and damage potential remain uncertain.
*Pandora's box*	Risks of this kind cause persistent, wide‐ranging, and irreversible changes without a clear attribution to specific damages, for example, persistent organic pollutant (CFCs caused the hole in the ozone layer) or endocrine disruptors.
*Cassandra*	These risks are relatively well known in terms of probability and damage potential, but there is a considerable delay between triggering event and the consequences, for example, climate change or loss of biodiversity.
*Medusa*	Risk of this type engenders a high potential of mobilization, although damage potential and probability are known as being low, such as electromagnetic fields.

Nowadays, most researchers agree that risk can be defined in statistical terms; it refers to the magnitude of losses or gains of an event multiplied by its probability of occurrence (Rosa, 1997). This probability‐oriented approach is helpful for decisionmakers in finding a way to balance the benefits of potential risk taking against the costs of risk aversion (Rothstein, Huber, & Gaskell, 2006). It allows them to convert fuzzy and controversial risks into precisely defined and relatively analytical entities (Stirling, 1998), which makes it possible to establish a relatively optimistic view of the possibilities of coping with risks (Lodge, 2009).

In contrast with the definitions of risk and uncertainty, Aven and Renn (2009) propose that “risk is defined as an event or a consequence in a certain setting: the consequences (outcomes) are uncertain and something of human value is at stake” (pp. 2–3). By not juxtaposing risk with uncertainty, Renn (2008), van Asselt (2005), and Aven and Renn (2010) identify four types of risk: *simple risk, uncertain risk, complex risk*, and *ambiguous risk*, based on characteristics of complexity, uncertainty^5^ and ambiguity.^6^ *Simple risks* refer to situations in which the causes of the risks are known, and there is no disagreement regarding the consequences or the interpretations of the risks (Renn, 2008). *Complex risks* imply that the causal reasoning between causes and effects are difficult to establish (Renn, 2008; van Asselt & Renn, 2011). In uncertain risks, we have difficulties in knowing the probability of occurrence of an event and its consequence beforehand because of ignorance or measurement errors (van Asselt & Vos, 2005, 2006). *Ambiguous risk* primarily refers to risks with value or norm ambiguity, implying that different actors may hold varied viewpoints or give different interpretations regarding the nature and consequences of the risks (Renn, 2008). The latter three types of risks are highly related to one another, and most risks are characterized by a blend of complexity, uncertainty, and ambiguity (Klinke & Renn, 2002, 2012; van Asselt & Renn, 2011; van Asselt & Vos, 2008).

Public policy scholars have also made contributions to the understanding of the nature of risk. Brown and Osborne (2013) have identified three different types of risk in relation to innovation: *consequential, organizational*, and *behavioral risk*. Consequential risk refers to risks in which the delivered services differ from established approaches, or new methods of doing things are tested. Organizational risk relates to the reputation and legitimacy of organizations, whereas behavioral risk refers to risks in the wider community.

Risk is a key issue in the planning and project management literature (Flyvbjerg, 2003; Guo, Richard, Wilkinson, & Li, 2014; Jaafari, 2001; Kutsch & Hall, 2010; Perminova, Gustafsson, & Wikstrom, 2008). Scholars argue that large projects inherently involve risks because of their complex interfaces, the involvement of multiple actors, and a lack of prior experience in dealing with large projects (Osipova & Eriksson, 2013). The Project Management Book of Knowledge defines project risk as “an uncertain event or condition that, if it occurs, has a positive or negative effect on at least one project objective, such as time, cost, scope, or quality” (Project Management Institute, 2000, p. 238). Based on current studies, we summarize seven main types of risk in planning and project management (Expert Group Report, 2010; Jaafari, 2001). They are presented in Table III.

**Table III:** Seven Types of Risk in Large Projects

Types of Risk	Description
*Market risks*	Found in both the supply and demand side. Demand risks refer to the situation in which markets are not large enough to justify investment, whereas supply risks refer to the risk that suppliers do not respond to the tenders because the specifications are too daring or too radical (Flyvbjerg, 2003).
*Technological risks*	Risks resulting in noncompletion, or under/false performance of a product. It could arise from choosing a suboptimal technology, a premature selection of a technology, or failure in recognition of technological incompatibilities (Jaafari, 2001).
*Financial risks*	These relate to the uncertainty of meeting target costs or securing the requisite funds. The development of innovative technologies always involves substantial economic investment. Flyvbjerg (2003) studied risk in relation to the megaprojects’ financial and economic aspects and established that it is difficult to estimate the target costs and safeguard funding for megaprojects due to many uncertain conditions, such as market conditions, economic crisis, or government policies.
*Environmental risks*	The possibility that large projects may lead to adverse environmental impacts beyond the permitted limits (Jaafari, 2001).
*Organizational risks*	Risks related to service delivery of public authorities. For instance, the adaptation of innovative technologies might require new skills or competencies of the administration. Governmental organizations may not, however, possess them (Expert Group Report, 2010).
*Social risks*	Risks in relation to a lack of acceptance by new users. Some innovative technologies have only been adopted for a short period, so we do not have wide‐ranging experience of them (Perrow, 1999). Consequently, some unanticipated events, such as strikes, riots, civil unrest or even wars, might occur. In the application of a waste incineration technique in China, citizens took to the streets to oppose its adoption, because they worried about its potential harmful effects on their health (Li, 2016).
*Turbulence risks*	Potential events that may significantly change the priorities of the involved actors and may result in unpredictable disastrous outcomes. Chernobyl is such an example, which led decisionmakers to rethink their policies in developing nuclear power plants (Aven & Renn, 2010).

In short, scholars from different academic fields and practical risk experts have different definitions of risk and risk assessment, analysis, and management. Existing definitions and understandings, categorizations and typologies are useful for researchers to understand the nature of risk. The aim of this contribution is the provision of a summary and synthesis of the existing literature and translating theory on risk and risk coping into risk coping strategies in public policy. To this end, we refer to the classical, widely spread understanding of risk as a mental construct by which to characterize potential hazards more precisely and to organize them according to the degree of threat, that is, cause–effect chains.

## 3 STRATEGIES FOR COPING WITH RISKS

Aven and Renn (2010) developed four different strategies for addressing risks: linear strategies, informed strategies, precautionary strategies, and discursive strategies. Linear strategy implies risk assessment agencies and formal institutions apply routine approaches to handle risks (van Asselt & Renn, 2011). Informed strategy is mainly applied with the aim of gaining more knowledge about risks through expert involvement (van Asselt & Renn, 2011). Precautionary strategy admits that it is impossible to eliminate uncertainties and recognizes we need to live/co‐exist with risks—but be prepared (Adam, 2002; Klinke & Renn, 2002; Todt & Lujan, 2014; Wynne, 1992). Discourse strategy recognizes that the process of managing risks is not linear. Rather, it is dynamic and iterative (van Asselt & Renn, 2011). It is essentially dialogue‐, collaboration‐, deliberation‐oriented (van Asselt, 2005), and its aim is to build consensus and resolve differences in values regarding the nature of the risks involved (Aven & Renn, 2012).

Some policy scholars have studied how uncertainties or complexities are addressed (Klijn & Koppenjan, 2016; Li, 2016; Lodge, 2009). Brown and Osborne (2013) have identified three generic strategies for addressing risks in innovation: *risk minimization strategy*, *risk analysis strategy*, and *risk negotiation strategy*. Risk minimization strategy refers to the avoidability of risk with the aim of eliminating risks. Risk analysis strategy essentially holds the assumption of predict‐and‐act, admits the inevitability of risk, and seeks ways to limit the consequences of it (Flemig, Osborne, & Kinder, 2016). It suggests that scientific knowledge is helpful in narrowing the supposed uncertainties and gaining a more precise definition of risk (Wynne, 1992). The risk negotiation strategy emphasizes the importance of interaction and collaboration among involved actors for coping with risks (Lodge, 2009). From a policy perspective, Nair and Howlett (2016) identify two types of policy design approaches for coping with uncertainties in climate change: the *robustness‐* and *resilience‐oriented* approaches. The robustness‐oriented policies aim at achieving stability within a specified range of uncertainty, whereas resilience‐oriented policies are designed with the aim of improving the adaptive capacities of systems through learning by doing and multi‐stakeholder participation (see also Huitema et al., 2016; van Buuren et al., 2013).

Some planning scholars consider two general strategies in planning and decision‐making processes: *expert involvement* and *the participatory approach* (Fischer, 2009; Fischer & Forester, 1993). The former means that experts are permitted to partake in the process of decision making and use their scientific knowledge and methods to obtain accurate information about the probability of accidents. The latter suggests that we need to integrate “science with participation” in addressing risks (Jasanoff, 1999), and the pragmatic experiential knowledge of different stakeholders about risk can be fused into scientific knowledge (de Marchi, 2003).

Scholars of project management have also contributed to the strategies for coping with risks in large projects (Atkinson et al., 2006; Chapman & Ward, 2003; Doloi, 2009; Koppenjan, Veeneman, van der Voort, ten Heuvelhof, & Leijten, 2011; Kutsch & Hall, 2010; Osipova & Eriksson, 2013; Rahman & Kumaraswamy, 2004; Turner, 2009). *Control‐* and *flexibility‐oriented* approaches are the two best known approaches in addressing risk in megaprojects (Osipova & Eriksson, 2013). The control‐oriented approach is essentially a top‐down approach for coping with risks, and its main aim is to predict risks. It places a high emphasis on the importance of planning, and it proposes that the planned project outcomes can be achieved through the rational identification and analysis of, and response to, risk (Lenfle & Loch, 2010). The flexible risk coping strategy is regarded as a relational partnership, implying that all parties should work together to jointly manage risks (Doloi, 2009; Geraldi, 2008; Koppenjan et al., 2011).

Researchers of complexity science also examine addressing risks in complex systems. Walker, Marchau, and Swanson (2010) identify three generic strategies in addressing unanticipated risks in complex systems: *resistance*, *resilience* 7, and *adaptation*. Resistance means that plans are made to prepare for the worst possible futures, whereas resilience aims at recovering quickly from them. Adaptation refers to changes of policy to accommodate new circumstances, implying that decisionmakers attempt to keep the system moving toward its original goal through monitoring and corrective action (McCray, Oye, & Petersen, 2010). The adaptation strategy encourages decisionmakers to consider “what if” situations and requires flexibility in the system to leave open options for coping with various plausible futures (Haasnoot, Kwakkel, Walker, & ter Maat, 2013; Walker et al., 2013).

Taleb (2012) studies how systems respond to unknown events. He develops a range of responses from *fragile*, *robust/resilience* to *antifragile*. *Fragility* refers to systems that are threatened by disorder, implying that the system suffers from the volatility and uncertainty of its environment. A fragile system is overoptimized because it is built to take account of its environment as stable and immutable (Abid et al., 2014), and it is built under the naïve assumption that uncertainties can be known (Taleb, 2012; White, 2013). Robustness or resilience refers to a system's ability of maintaining a desired state when exposed to a range of stresses. If a system can maintain stability over a long range of stresses, it is considered robust (Kennon, Schutte, & Lutters, 2015; Platje, 2015). In contrast with fragility, a robust^8^ system can withstand or absorb pressures. It resists shocks and stays the same (Taleb, 2012). Antifragility means that a system grows stronger as a result of each successive failure and disturbance (Taleb, 2012). It allows a system to move away from a predictive mode of thinking toward a mode that embraces uncertainty, chaos, volatility, variation, and randomness (Aven, 2015; Gorgeon, 2015). Antifragility wants failure to be a nonevent (something that runs all the time in the background), so that when a real failure occurs, it can be handled without any impact (Abid et al., 2014).

In short, our literature review reveals ample knowledge about the topic of risk coping strategies. Different types of coping strategies in addressing risks from various academic fields were identified. Some of them share similarities to some degree. For instance, the flexibility strategy identified in project management literature is akin to the risk negotiation strategy identified by policy scholars and the antifragility strategy identified by Taleb (2012). Although these concepts have been used by scholars from different academic fields, they share some general consensus regarding their implications on risk coping. However, we also recognize that scholars from different fields at times have used the same concept with different interpretations and meanings. For instance, the term resilience has the same meaning as the term robustness in Taleb (2012). However, Nair and Howlett (2016) view robustness and resilience differently. The former mainly refers to the stability, whereas the latter refers to adaptive capacities. Moreover, the concept resilience and adaptation sometimes are defined differently. In their in‐depth study of the terms associated with “flexibility” in relation with infrastructure systems, De Haan, Kwakkel, Walker, Spirco, and Thissen (2011) examined 11,029 article titles and abstracts and identified a number of associated terms. De Haan et al. (2011, p. 926) first proposed the following definitions:

1. *Adaptive infrastructure constellation*: “…can be altered to keep on meeting a societal need under changed circumstances.”
2. *Resilient infrastructure constellation*: “…can resume meeting a societal need under changed circumstances.”
3. *Robust infrastructure constellation*: “…can keep on meeting a societal need under changed circumstances.”

Afterward they applied a data mining approach and brought forward the following conclusions about the terms adaptivity, resilience, and robustness and their comparisons in the context of infrastructure constellations. Their study highlighted that adaptivity predominantly relates to changes on the longer term and is associated with the ability to change along with circumstances focusing on recovery, after the fact, rather than to anticipate change. Resilience is often associated with recovering from shocks and disturbances and bouncing back from them on short timescales while robustness is similar to adaptability predominantly related to longer timescales and is indifferent with regards to anticipatory action or recovery.

In this contribution, our main ambition is to reconstruct different categories, understandings, and topologies in an aggregated level. Many detailed debates among scholars about the definitions of risk and risk coping strategies are not the focus of this contribution. We synthesize various concepts in a broad, or generic, but generally acceptable manner to formulate a unified conceptual framework for facilitating future empirical studies and risk practices in public policymaking. We appreciated the work conducted by scholars who have addressed and discussed the differences between risk, complexity, uncertainty, and ambiguity (Aven & Renn, 2010; Klinke & Renn, 2002, 2012, 2014; Stirling, 1998; 2007), and differences between flexibility, resilience, robust, and adaptation (De Haan et al., 2011). These studies are helpful and instructive for researchers to clarify the meaning of these terms.

Moreover, scholars from different academic communities exchange ideas and create many fresh insights into the issue of risk management. For instance, studies on complex technological systems provide scholars from other fields with insights into the nature of risk (Gerrits, 2016; Perrow, 1999). Planners and decisionmakers initially believed that risks involved in complex systems were controllable and predictable, and they designed systems with the aim of surviving all potential threats and dangers. Increasingly, they recognized that the components of the complex technological systems interact in nonlinear ways, and the properties of complex sociotechnical systems cannot be well understood (Byrne, 1998). A small change at a certain threshold may also result in cascading outcomes, just as adding another grain of sand to the pile at some indeterminate point causes the entire sandpile to rearrange itself without outside intervention (Comfort, 1994). Finally, planners and decisionmakers admit that there are always uncertainties waiting to happen and it is impossible to control them fully (Perrow, 1999). The ideas of complexity science are thus helpful for policy scholars in acknowledging the inherent unpredictability of risks.

## 4 REFRAMING RISK COPING STRATEGIES FOR PUBLIC POLICY

After reviewing the various strands of literature, we identify one major issue in the existing scope and variety of risk coping strategies in terms of problem‐solving capacity in public policymaking: there is no widely accepted conceptual frame for policy scholars to grasp the spectrum of risk coping strategies as a coherent whole. Some conceptual frameworks established by researchers from different disciplines and domains, such as planning, project management, risk governance and research on resistance, resilience, and adaptation, are useful in helping us to identify possible options for any involved actors in addressing risks. We nevertheless assert that, first, public policy is lacking a critical review and thorough summary of risk coping strategies or risk management strategies, and, second, the findings in other fields cannot be directly applied by decisionmakers in governing risks.

An important task of public policy is that administrative executive branches of the state determine, promulgate, and perform adequate and rational coping strategies concerning a given risk or a class of risk issues. Public policy studies reveal that the quality of government strategies in managing risk plays a crucially important role in influencing the daily life of citizens. Designing risk coping strategies and respective actions and regulatory measures as rational and satisfactory in quality and quantity does not only presuppose the evaluation of risk, but it also has the effect of evaluating certain kinds of actions or behavior as superior to others. Furthermore, it justifies public policy grounded in assumptions about what constitutes rational individual or collective action or behavior in view of risk. Governments are often guided by values and motives that are distinct from other actors and their preferences in addressing risk. Public policy often tends to be governed by instrumental reasoning, that is, achieving the most efficient means to some desired end. As such, the right risk coping strategy may be biased against the standard manner of government action and processes. Each risk or class of risk is influenced by different problems and concerns and has different stakeholders. Numerous agencies, corporations, nonprofit organizations, interest groups, risk experts, and laypeople compete and collaborate to influence decisionmakers to select the right risk coping strategy or a set of strategies. Although many actors are relevant in addressing risk, government officials ultimately choose risk coping strategies in public policy‐making processes that are deemed to solve the risk problem at hand and expected to meet the common good. In this article, we have reviewed the emerging scope and variety of coping strategies and have considered their nuances. As emphasized throughout, the field of problem‐solving strategies addressing risk has become diversified and it is difficult to describe in general and disjunctive terms because different strategies have been put forth by various strands of social science literature. In conclusion, this section recapitulates the reviewed literature by identifying and highlighting a basic pattern of problem‐solving strategies addressing risk from a public policy perspective. We outline six archetypes of problem‐solving strategy addressing risk in public policy that represent a kind of continuum: *no response, prevention‐, control‐, toleration‐, precaution‐, and adaptation‐oriented* strategies. It is worth mentioning again that we are primarily interested in state entities because they play a pivotal role in coping with risks and coordinating the relationships between the different stakeholders involved.

1. *No response*: No specific actions are taken by decisionmakers for addressing risks. This strategy partially corresponds with the fragility strategy proposed by Taleb (2012) and Duit and Galaz (2008). It can be identified through several indicators. First, decisionmakers may repeatedly put off decisions because of the uncertainty of the risks involved. For example, many countries put off decisions regarding climate change, given the uncertain nature of climate change (Adger, Lorenzoni, & O'Brien, 2009). No response also implies that no back‐up plans are developed by decisionmakers for addressing impending dangers and threats. For instance, if a country in a region has a narrow economic basis (such as agriculture or mining), then it is fragile to uncertain economic situations (Sorensen, 2015). Finally, the no response strategy implies that no routine institutions are established to address risks. One example is that a developing country may have limited institutional capacities to maintain stability. When crises come, the whole country tends to collapse (Duit & Galaz, 2008). No response to risks tends to result in substantial negative consequences for populations, states, and ecosystems (Walker et al., 2010). There might also be a rational argument: If you are convinced that waiting will reduce costs because you do not invest in ineffective measures it may be better to wait for a response until more clarity is given.
2. *Prevention‐oriented strategy*: Decisionmakers take preventive actions with the aim of avoiding risks, such as building a wall to prevent the invasion of enemies (Longstaff, 2005). This strategy corresponds with the risk minimization strategy proposed by Brown and Osborne (2013) and (partially) with the linear strategy proposed by Aven and Renn (2010). Essentially, the main aim of this strategy is *risk avoidance*. One example of this strategy is that decisionmakers may prohibit the adoption of innovative technologies to prevent the occurrence of risks. This strategy is appropriate for situations in which changes are highly predictable (Wildavsky, 1991), and it is possible to promote coordination and reduce discretion and facilitate predictability. It has slow responsiveness, however, and the designed systems tend to become paralyzed or self‐destructive in the face of unexpected dangers and threats (Comfort, 1994).
3. *Precaution‐oriented strategies*: These often rely on the EU's accentuation of two significant aspects in developing and implementing public policy applying the precautionary principle (Klinke, Dreyer, Renn, Stirling, & van Zwanenberg, 2006; see also Adams, 2002; Goklany, 2001; O'Riordan & Cameron, 1994). First, precaution‐oriented strategies are taken into account within a risk analysis framework consisting of risk assessment, risk management, and risk communication. In the risk assessment process, a scientific evaluation is to be completed that identifies and quantifies, if possible, the degree of scientific uncertainty. Second, since a wide range of risk management instruments is possible, the action and measures taken in precaution‐oriented strategies presuppose transparency, proportionality to the chosen level of protection, nondiscrimination in their application, and consistence with similar measures that have been previously taken. Furthermore, precaution‐oriented public policies usually also include cost–benefit examinations, reviews in the light of new scientific data and the capability of assigning responsibility for producing the scientific evidence necessary for a more comprehensive risk assessment.
4. *Control‐oriented strategy*: Efforts are made primarily with the aim of controlling risks. This strategy corresponds with the informed strategy proposed by Aven and Renn (2010), the risk analysis strategy proposed by Brown and Osborne (2013), the control strategy proposed by Osipova and Eriksson (2013), and the expert involvement strategy proposed by Fischer (2009). This strategy emphasizes centralization and “high modernist” forms of surveillance (Lodge, 2009), and it partially corresponds with our understanding of the regulatory state, in which decisionmakers dominate the processes of risk management and they attempt to regulate risks through formal policies, laws or regulations (Moran, 2003). An example of this strategy is that decisionmakers apply existing policies to regulate the debated issues with the aim of controlling risks (Witt, Suzor, & Wikstrom, 2015). In contrast with the prevention‐oriented strategy, the control‐oriented strategy essentially allows for the existence of risks and attempts to predict and regulate rather than eliminate them. The difference between precaution‐oriented strategy and the control‐oriented strategy is that in the precaution‐oriented strategy actions are taken under high degree of uncertainty when the likelihood of the events cannot be determined while their consequences could be high. In contrast, control‐oriented strategy is highlighting the traditional actions of the governments and the de facto command and control functions they perform under normal conditions. In control‐oriented strategy, actions taken are to address risks that are well understood through risk assessment and corresponding regulations, policies or laws.
5. *Toleration‐oriented strategy*: Decisionmakers take action to prepare for risks with the aim of enabling a system or organization to perform satisfactorily in a wide range of situations. This corresponds with the resistance strategy proposed by Walker et al. (2013), the robustness strategy proposed by Nair and Howlett (2016), and the robustness/resilience strategy proposed by Taleb (2012). Developing alternatives is the first option of this strategy (Landau, 1969). In the energy provision market, governments prepare several different sources of energy for impending unanticipated events; when one source of energy is unavailable, the other sources of energy can be alternative options (Longstaff, 2005). Another example in the field of supply chain management occurred in 2000, when one of Nokia's key cell phones suffered a major fire. Nokia identified this crisis quickly, secured alternative supplies and modified its product design to broaden its sourcing options (Fiksel, Polyviou, Croxton, & Pettit, 2015). This strategy also means that policy changes or reforms to mitigate the potential consequences are prepared in advance (Walker et al., 2010).
6. *Adaptation‐oriented strategy*: It refers to efforts in promoting the adaptive capability of the systems. This corresponds with the idea of adaptive resilience proposed by Boin and van Eeten (2013), Duit (2016), and Nair and Howlett (2016), the resilience strategy proposed by Walker, Haasnoot, and Kwakkel (2013), the risk negotiation strategy proposed by Brown and Osborne (2013), the flexibility strategy proposed by project management scholars (Osipova & Eriksson, 2013), the participatory strategy proposed by Fischer (2009), and the antifragility strategy proposed by Gorgeon (2015) and Taleb (2012). This strategy is characterised by several options, for example, decentralization, self‐organization, forward‐looking planning, joint responsibility, learning by doing, deliberation and participation, and co‐deciding (Adger, Lorenzoni, & O'Brien, 2009; Fischer, 2009; Huitema et al., 2016; Li, Koppenjan, & Verweij, 2016; Lodge, 2009; Nair & Howlett, 2016; Taleb, 2012; van Buuren et al., 2013). This strategy has been adopted in many different fields such as flood risk management, innovative urban transport infrastructure, the expansion of ports, and energy projects (Marchau, Walker, & van Duin, 2008; Marchau, Walker, & van Wee, 2010). Another example is that Dutch water managers were required to update their response plans with the release of new climate scenarios. The final plans were not dependent on the climate scenarios available at the time, but required a forward‐looking design for various possible future scenarios (Delta Programme Commissioner, 2014).

Compared with the other works, our conceptual framework has two advantages. First, it is comprehensive, showing the variety of strategies for coping with risks. It widely borrows insights from the other conceptual frameworks and shows the nuances of different government actions in responding to risk (see column 2, Table III). Several different strands of literature have been reviewed, enabling us to encompass the whole continuum of strategies that can be applied by decisionmakers for addressing risks. This makes it possible to provide a comprehensive framework specifically for scholars in public policy. Second, our conceptual framework is operationalizable. Many conceptual frameworks established by researchers cannot be widely applied in empirical studies, because many concepts are difficult to operationalize. Our conceptual framework attempts to resolve this issue, and indicators are identified to facilitate the operationalization of different risk coping strategies. For instance, putting off decisions in coping with risks indicates the emergence of a no response strategy. The operational definitions of the six risk coping strategies, their connections with the strategies of the others, and the operational indicators are presented in Table IV.

**Table IV:** Six Types of Government Strategies for Coping with Risks

Type of Government Strategy	Examples of Connections with Other Studies	Key Dimensions	Indicators
No response	Fragility (Taleb, 2012), and fragile strategy (Duit & Galaz, 2008)	No action	Putting off decisions No back‐up plans Absence of routine institutions
Prevention‐oriented	Prevention (Longstaff, 2005) and linear approach (Aven & Renn, 2010)	Prohibition	Preventing or banning
Precaution‐oriented	Based on precautionary principle (Klinke et al., 2006; O'Riordan & Cameron 1994; Goklany, 2001; Adams, 2002)	Precautionary action	Risk assessment, management, and communication Application of precautionary principle
Control‐oriented	Informed strategy (Aven & Renn, 2010), risk analysis (Brown & Osborne, 2013), control strategy (Osipova & Eriksson, 2013), expert involvement (Fischer, 2009)	Control and regulation	Risk analysis Expert involvement Regulation
Toleration‐oriented	Resistance strategy (Walker et al., 2013), robustness strategy (Nair & Howlett, 2016), robustness/resilience (Taleb, 2012)	Reform and creation of alternatives	Development of alternatives Reform of existing regulations
Adaptation‐oriented	Adaptive resilience (Boin & van Eeten, 2013; Duit, 2016; Nair & Howlett, 2016), resilience (Walker et al., 2013), risk negotiation (Brown & Osborne, 2013), flexibility strategy (Osipova & Eriksson, 2013), participatory strategy (Fischer, 2009), and antifragility strategy (Taleb, 2012)	Collaboration and negotiation	Decentralization Learning by doing Co‐deciding/negotiation/deliberation^a^ Forward‐looking planning

^a^These indicators are all in favor of a collaborative approach to address risk, implying all stakeholders should be involved to build a consensus regarding the nature of risks they face and the strategies for addressing those risks. The consequences resulting from risks should be taken on board by all stakeholders.

## 5 CONCLUSION AND IMPLICATIONS

This article has reviewed and analyzed the evolution and scope of risk coping strategies over the last two decades from a public policy perspective and provided a commonly shared understanding among scholars with regard to risk problem‐solving practices as applied by governments. We began by introducing background information about the definitions of risks and risk coping strategies to increase our understanding of risk management. We found that scholars have knowledge about the definition of risk and strategies for addressing risk, but we recognize that scholars in public policy have not established a comprehensive framework to grasp the spectrum of strategies that are specifically applied by governments for coping with risk. We have therefore reconstructed and reframed the entire range of existing risk coping strategies, consisting of six governmentally initiated problem‐solving options: no response, prevention‐oriented, control‐oriented, precaution‐oriented, toleration‐oriented, and adaptation‐oriented strategy. It should be noted that the concepts we presented as such were not new: they aim to serve as a heuristic to understand and summarize practices in applying risk coping strategies from a public policy perspective.

In addition, we recognize that current studies are mostly theory‐oriented and focus primarily on the definitions, features and typologies in relation to risk and risk coping. Only a few empirical studies have investigated risk coping strategies. The conceptual framework established in this contribution can inform future empirical research in public policy on risk coping strategies, both for case study research and cross‐national comparison, and can facilitate risk management practices. A small number of applications have already been published using this framework to analyze the governance of risks in ridesharing and autonomous vehicles (Li, Taeihagh, & Jong, 2018; Rosique, Navarro, Fernández, & Padilla, 2019; Taeihagh & Lim,^2019^).

In the following, we would like to formulate a research agenda to further enhance the applicability of our framework and enhance the field of risk governance from a public policy perspective.

First, more in‐depth case studies could be conducted to explore and evaluate the applicability of our reframed spectrum of risk coping strategies as an analytical template. Take the management of risk in innovative technologies as an example. Many innovative technologies (such as big data, open data, crowdsourcing, and the Internet of things) are increasingly being adopted by different countries and cities around the world. Substantial opportunities are available to investigate how governments cope with risk in relation to the adoption of these technologies. One plausible option is to conduct case studies to research how a single government addresses the risks involved in taking on a specific type of innovative technology. For example, it is possible to examine how a single government (such as that of the United Kingdom, Singapore, or China) copes with the risk involved in adopting autonomous cars. Another option is to conduct comparative case studies to explore how a single government copes with innovative technologies from different sectors (such as energy, ICT, waste, transport, and water), or how different governments cope with the risks associated with the same technology. For example, we can compare how the U.S. government deals with the risks involved in the adoption of solar energy and waste incineration. We can also compare how risks associated with ridesharing are governed by different governments around the world (such as China and Singapore). Through these single and comparative case studies, we can examine whether our conceptual framework can be used in categorizing different government responses to the risk involved in adopting innovative technologies.

Second, our conceptual reframing could be further developed through providing explanations about the choices of strategies for coping with risks. This article provides us with a problem‐solving taxonomy used to classify risk coping strategies that helps us identify and understand governmentally driven public policy strategies in addressing risks. It does not, however, help explain the choice of strategies in addressing risks. In the next stage, it is feasible for researchers to identify which factors lie behind a government's choice of strategy. One possible option is comparative and evaluative analyses to draw implications and conclusions in terms of similarities and dissimilarities of different cities/countries when applying different risk coping strategies on the same risk phenomena and their related reasoning processes.

Third, researchers can explore how a system or organization can adapt to risks under uncertain conditions. Researchers from different academic communities have told us that adaptation is a better strategy for responding to risks than the traditional resistance or prevention strategy (Nair & Howlett, 2016; van Buuren et al., 2013). It is, however, still unclear what the mechanisms for adaptation are. Future studies are needed to investigate the conditions under which precaution or adaptation will emerge and/or last.

## DISCLOSURE STATEMENT

The authors declare no conflict of interest.

## Footnotes

1 To arrive at the literature review below, we first gathered and analyzed our findings regarding the nature of risks and the risk coping strategies in their chronological order of appearance. We then consulted various risk governance experts to ensure we included all classic references. Finally, since this article aims to make a contribution to scholarship in public policy, we focused our review on domains highly related to public policy, such as planning, governance and public policy, crisis management, and complexity. Based on these principles, we arrived at the examples and models that show up in this overview of the theory.

2 Level 1 uncertainty refers to a clearly predictable future, which represents a situation that is not absolutely certain but of an explicitly measurable degree of uncertainty. Level 2 uncertainty refers to alternative futures with probabilities; it implies that the uncertainty can be measured in statistical terms and it can either be a single future with a confidence internal, or multiple features with various likelihoods. Level 3 uncertainty refers to alternate futures that can be ranked. This means that multiple alternatives can be computed and ranked with regard to the perceived likelihood. Level 4 uncertainty refers to a situation in which a multiplicity of plausible futures can be enumerated, but the alternatives cannot be ranked based on the perceived likelihood. Level 5 uncertainty refers to the unknown future. It is the deepest level of uncertainty and is characterized as ignorance. It corresponds with Taleb's (2007) understanding about black swans, which refers to events that are beyond our expectations.

3 Researchers offer different definitions of ambiguity. Jensen and Wu (2016) view ambiguity as one type of uncertainty, because multiple actors involved have different interpretations of the information they obtain. Similarly, Brugnach and Ingram (2012) argue that ambiguity refers to the presence of several valid but sometimes contradictory frames about a problem.

4 This point is shared by Wildavsky (1991), who views uncertainty as the knowledge that events will take place, but not of their probabilities.

5 Unlike for Stirling (2007), uncertainty here implies that the potential probabilities of a specific event are unclear, but there is agreement on its outcomes. Here, uncertainty means that we have difficulties predicting the occurrences of an event and its consequences due to ignorance or measurement errors.

6 Unlike Stirling (2007), ambiguity does not mean a situation in which probabilities are known but outcomes are unknown. Here, ambiguity implies that various actors have different values regarding the question of whether risks are acceptable or tolerable. It means “there are different legitimate viewpoints from which to evaluate whether there are or could be adverse effects and whether these risks are tolerable or even acceptable” (van Asselt & Renn, 2011, p. 437).

7 Understanding of the concept of resilience differs and can be generally distinguished into three categories: *precursor resilience*, *recovery resilience* and *adaptive resilience* (Boin & van Eeten, 2013; Duit, 2016). Precursor resilience is defined as the capacity to prevent problems from becoming disastrous crises, implying that a system can absorb risk and maintain its function when accidents come (Boin & van Eeten, 2013; Taeihagh, 2017). Recovery resilience refers to the capacity to return back to a normal state. An organization or a city rising from the ashes of a crisis is an example of recovery resilience. Adaptive resilience prioritizes learning, lesson‐drawing, and institutional and organizational changes (or reforms) to increase resilience in the future. It corresponds with the understanding of ecological resilience, which originates from a socioecological systems approach. This primarily involves adaptation, flexibility, learning, and redundancy (Folke, 2006). These different understandings of resilience can lead to confusion. Most scholars tend to adhere to the etymological definition of resilience: *to maintain stability and return* (Wildavsky, 1991). The literature overwhelmingly emphasizes the positive image of resilience (Boin & van Eeten, 2013), but resilience may be harmful if it lets negative conditions persist, for example terrorist organizations (Bakker, Raab, & Milward, 2012).

8 Here, Taleb (2012) equals robustness with resilience. However, Capano and Woo (2017) argue that resilience and robustness are not necessarily related and are conceptually distinct. Broadly speaking, robustness is the “ability to withstand or survive external shocks, to be stable in spite of uncertainty” (Bankes, 2010, p. 2), and resilience refers to the capacity of recovery form external shocks (Wildavsky, 1991).
